# Complete Genome Sequence of the Tataguine Virus, Isolated in the Central African Republic in 1972 from a Human with an Acute Febrile Syndrome

**DOI:** 10.1128/MRA.01248-18

**Published:** 2019-02-21

**Authors:** Huguette D. Simo Tchetgna, Benjamin Selekon, Mirdad Kazanji, Nicolas Berthet, Emmanuel Nakoune

**Affiliations:** a Centre Interdisciplinaire de Recherches Médicales de Franceville (CIRMF), Franceville, Gabon; b Institut Pasteur de Bangui, Bangui, Central African Republic; c Institut Pasteur, Unité Environnement et Risques Infectieux, Cellule d’Intervention Biologique d’Urgence, Paris, France; d Centre National de Recherche Scientifique (CNRS) UMR3569, Paris, France; Broad Institute of MIT and Harvard University

## Abstract

Tataguine virus (TATV) is an orthobunyavirus that causes febrile illnesses in Africa. Here, we report the complete genome sequences of TATV strain HB72P583, isolated in the Central African Republic in 1972. Several genetic variations were detected in the small (S), medium (M), and large (L) segments relative to a TATV strain isolated in Nigeria in 1966.

## Abstract (precis)

Tataguine virus (TATV) is an orthobunyavirus that causes febrile illnesses in Africa. Here, we report the complete genome sequences of TATV strain HB72P583, isolated in the Central African Republic in 1972.

## ANNOUNCEMENT

The tataguine virus (TATV) belongs to the *Orthobunyavirus* genus in the *Peribunyaviridae* family (1). Following its initial isolation from *Anopheles* and *Culex* mosquitos in 1962, TATV has been implicated in several cases of febrile illnesses throughout sub-Saharan Africa, mainly in Senegal, Nigeria, Cameroon, Ethiopia, and the Central African Republic (1, 2). The virus is thought to be transmitted by the malaria vectors Anopheles gambiae, Anopheles funestus, and Anopheles nili (3).

Here, we describe a TATV strain, HB72P583, isolated in 1972 in the Batangafo region of the Central African Republic (CAR) from a human presenting dengue-like symptoms. This virus was isolated and amplified by serial passages in newborn mouse brains. Supernatants of homogenized brain tissues were lyophilized and stored in sealed glass vials at room temperature until 2012. Viral genomic RNA material was extracted from lyophilizates that were resuspended in phosphate-buffered saline using a QIAamp viral RNA minikit (Qiagen, Germany). The extracted RNA was retrotranscribed into cDNA using SuperScript III enzyme and random hexamers (Invitrogen Corporation, Carlsbad, CA). This cDNA was then used for a whole-transcriptome amplification (WTA) step (QuantiTect whole-transcriptome kit, Qiagen) based on Phi29 polymerase as described previously (4). Amplified DNA was fragmented by ultrasonication according to the manufacturer’s instructions (Covaris M220, Covaris Inc., USA). The generated DNA fragments were used to construct a genomic library with the NEBNext Ultra DNA library prep kit for Illumina (New England Biolabs) according to the manufacturer’s recommendations. The Illumina sequencing was performed using a HiSeq 2000 sequencer to obtain paired-end reads of 100 bp. Read quality control and trimming were performed using FastQC version 0.11.6 (Babraham Bioinformatics) with default parameters. After the quality control analysis, all the trimmed reads were aligned to the Mus musculus Mn10 reference genome. All the reads corresponding to this species were then discarded, while the unaligned reads were recovered to move forward with the analyses. Then all the reads corresponding to viral sequences were obtained based on a “similarity-based” approach using BLASTN (evalue: 0.0001 and output format [outfmt] with the flag 6) with publicly available sequences (GenBank accession numbers KY555802, KY555803, and KY555804). To improve the assembly quality of the viral genomic segments, only the regions of each read matching BLAST results were selected and conserved (5). Finally, all the conserved regions of each read were assembled using SPAdes version 3.5 with variable k-mer values to build the De Bruijn graph and generate the three genomic segments (6).

We obtained three contigs of 911, 4,420, and 6,816 bp corresponding to the small (S), medium (M), and large (L) genomic segments, respectively. Genomic analysis reflected the typical organization of a *Peribunyaviridae* genome. Our sequence shows, respectively, 16, 79, and 97 single nucleotide polymorphisms (SNPs) in the S, M, and L segments with respect to the 1966 Nigerian reference genome IBH10362 (GenBank accession numbers KY555804 [S], KY555803 [M], and KY555802 [L]). Of these SNPs, only 14, 76, and 95 were in the coding regions of nucleoprotein, polyprotein, and RNA polymerase, respectively. Finally, there were only 3, 26, and 11 amino acid changes in the nucleoprotein, polyprotein, and RNA polymerase sequences, respectively.

In conclusion, given the recent global emergence of arboviruses and their public health consequences, it is important to be able to better understand the circulation of the TATV virus in its *Anopheles* vectors and its burden on febrile illnesses in Africa.

### Data availability.

The complete genome sequences of TATV strain HB72P583 were deposited in GenBank under the accession numbers MH603134, MH603135, and MH603136.
